# Effects of Naltrexone on Pain Sensitivity and Mood in Fibromyalgia: No Evidence for Endogenous Opioid Pathophysiology

**DOI:** 10.1371/journal.pone.0005180

**Published:** 2009-04-13

**Authors:** Jarred W. Younger, Alex J. Zautra, Eric T. Cummins

**Affiliations:** 1 Department of Anesthesia, Stanford University School of Medicine, Palo Alto, California, United States of America; 2 Department of Psychology, Arizona State University, Tempe, Arizona, United States of America; 3 College of Medicine, University of Arizona, Tucson, Arizona, United States of America; University of Otago, New Zealand

## Abstract

The pathophysiological mechanisms underlying fibromyalgia are still unknown, although some evidence points to endogenous opioid dysfunction. We examined how endogenous opioid antagonism affects pain and mood for women with and without fibromyalgia. Ten women with fibromyalgia and ten age- and gender-matched, healthy controls each attended two laboratory sessions. Each participant received naltrexone (50mg) at one session, and placebo at the other session, in a randomized and double-blind fashion. Participants were tested for changes in sensitivity to heat, cold, and mechanical pain. Additionally, we collected measures of mood and opioid withdrawal symptoms during the laboratory sessions and at home the night following each session. At baseline, the fibromyalgia group exhibited more somatic complaints, greater sensory sensitivity, more opioid withdrawal somatic symptoms, and lower mechanical and cold pain-tolerance than did the healthy control group. Neither group experienced changes in pain sensitivity due to naltrexone administration. Naltrexone did not differentially affect self-reported withdrawal symptoms, or mood, in the fibromyalgia and control groups. Consistent with prior research, there was no evidence found for abnormal endogenous opioid activity in women with fibromyalgia.

## Introduction

Fibromyalgia is a chronic and debilitating condition, characterized primarily by diffuse musculoskeletal pain, mechanical allodynia, and hyperesthesia [1]. Other symptoms–including sleep disturbance, fatigue, anxiety, depression, and cognitive impairment–further contribute to its severity [2], [3]. Fibromyalgia affects approximately 5% of women [4] and 1.6% of men [5] in the general U.S. population, many of whom still need effective treatment options [6]. The condition significantly impacts the quality of life for affected individuals [7] and imposes a large economic burden on society [8].

Recent studies of fibromyalgia pathophysiology point to central nervous system dysregulations [9]. One central nervous system target of investigation has been endogenous pain modulators [10], [11] such as opioid-peptides [12]. We focused on beta-endorphin, which is an important mediator of analgesia [13]. Previous studies that contrasted peptide concentrations in fibromyalgia and control groups found no differences in cerebrospinal fluid [14] or blood plasma [15], [16]. However, one study identified lower concentrations of beta-endorphins in the peripheral blood mononuclear cells of fibromyalgia patients [17]. A recent study also found decreased mu-opioid receptor availability in the brains of fibromyalgia patients [18]. Despite the indications of altered mu-opioid systems, three studies employing an experimental design and the (predominantly *mu*-) opioid receptor antagonist naloxone failed to find any significant effect in fibromyalgia. Two of those studies found that naloxone did not affect pain sensitivity [19], [20], and the third concluded that naloxone's effects are similar to those of a placebo [21].

To help address the conflicting information in the literature regarding endogenous opioid peptide involvement in fibromyalgia pathophysiology, we conducted this study on the effects of opioid antagonism in fibromyalgia pain and mood symptoms. We examined two *competing* hypotheses of how groups might differ in their responses to naltrexone. In Hypothesis 1, we predicted that women with fibromyalgia would be less affected by naltrexone administration than healthy controls (as represented by smaller increases of pain sensitivity and negative mood under naltrexone). Hypothesis 1 corresponds with the view that women with fibromyalgia have deficient endogenous pain inhibition. In the competing Hypothesis 2, we predicted that women with fibromyalgia would show exaggerated increased pain sensitivity and negative mood when given naltrexone, as compared to healthy controls. Hypothesis 2 represents the view that although opioid-based pain systems are maximally engaged in fibromyalgia, they insufficiently attenuate pain [22].

## Methods

### Study overview

In our study, women with and without a diagnosis of fibromyalgia attended two laboratory sessions, which were held on separate days. Participants received the opioid antagonist naltrexone in one session, and placebo in the other. Drug administration was randomized and double-blinded. In each laboratory session, tests of pain sensitivity were administered, as well as measures of mood and opioid withdrawal. We extended others' experimental drug methodology [19], [21] in a few important ways: 1) Both physical and psychological manifestations of fibromyalgia were measured. 2) The more potent opioid receptor antagonist naltrexone was used instead of naloxone. 3) Symptoms directly associated with opioid withdrawal were recorded. 4) Multiple pain modalities were used, including heat, cold and mechanical. 5) Home measurements were collected on some variables the night following each laboratory session.

### Participants

Participants were 10 women with fibromyalgia and 10 healthy controls matched for gender, age, and income, whose average age was 55 years (*SD* = 7.7). Most of the participants were Caucasian (85%); two were Hispanic, and one was African-American. The average daily pain level for women with fibromyalgia was 6.1 on a 10-point scale (standard deviation of 2.4), a score that indicates moderately-high disease severity. Subjects were recruited via flyers or newspaper announcements in the greater metropolitan area of Phoenix, Arizona. Inclusion criteria included: 1) 18 or more years of age, 2) ability to give informed consent, and for the fibromyalgia group, 3) a physician's diagnosis of fibromyalgia. Exclusion criteria were: 1) current or past use of opioid medications and 2) pregnancy, or plans to become pregnant. All participants completed an evaluation for fibromyalgia on their first study visit, to confirm the physician diagnosis. Participants were fully informed of all study procedures and the risks involved, and all procedures were approved by the Institutional Review Board at Arizona State University. Written consent was obtained at the first laboratory visit.

In the fibromyalgia group, two women were taking prescription medications for pain (celecoxib, cyclobenzaprine, and carisoprodol), two were taking antidepressant or anxiolytic drugs with potential analgesic effects (trazadone and sertraline), and two were taking sleep-aid medications as needed (eszopiclone and zolpidem). One woman in the control group was taking an antidepressant (paroxetine). Participants were allowed to continue their medication throughout their participation in the study.

### Measures

#### Baseline descriptive measures

To determine baseline differences between the two groups, three measures were completed before any capsules were administered: fibromyalgia severity, general somatic complaints, and sensitivity to sensory stimulation. In both groups, we administered the 10-item Fibromyalgia Impact Questionnaire (FIQ; [23]) that measures severity of fibromyalgia. The FIQ is a widely used scale with good reliability and validity [24]. General somatic complaints were measured with the Somatization and Emotional Contribution Scale (SECS). The SECS is an author-generated, original somatic symptom checklist of 42 commonly experienced physical symptoms such as headache, muscle tension, and dizziness. SECS exhibits good internal consistency (α = 0.93) and construct validity [25] with the Somatization sub-scale of the Symptom Checklist-90R [26]. Sensitivity to sensory stimulation was assessed with the Sensory Hypersensitivity Scale (SHS; [27]). The SHS a 25-item scale that can assess general sensitivity (internal reliability of α = 0.80) or modality-specific sensitivity (taste, light, sound, smell, pain, heat, cold, texture, and allergies). For the present analyses, we examined only the general sensitivity scores.

#### Naltrexone outcome measures

Drug response was assessed via three quantitative sensory tests of pain. The three pain tests were: 1) heat pain threshold and tolerance, 2) cold pain threshold and tolerance, and 3) mechanical pain threshold. To measure heat pain threshold and tolerance, a thermode was placed on the thenar eminence of the hand and heated from a baseline temperature of 32°C at a rate of 0.5°C/sec (maximum 50°C). Participants indicated via pressing a button when the temperature first became painful (threshold) and when the heat was no longer bearable (tolerance), at which point the thermode immediately cooled. Cold pain threshold and tolerance were measured following a similar protocol to heat. The thermode was placed on the thenar eminence of the opposite hand and cooled down from 32°C at a rate of 0.5°C (minimum 0°C). Participants indicated both pain threshold and tolerance levels. Mechanical pain sensitivity was measured using the 18 sites defined by the American College of Rheumatology [28]. Pressure was applied to the sites using a Fischer dolorimeter (Pain Diagnostics, Great Neck, NY) and 1 cm^2^ rubber disk, at a rate of 1 kg/cm^2^/s, until the participant indicated pain. Pain threshold was recorded in kg/cm^2^, and all points were averaged to yield a total score of mechanical pain threshold. Some studies have found mechanical stimulation to be superior to thermal stimulation in predicting clinical pain [29], [30].

In addition to the pain tests, participants also completed two self-reported questionnaires which examined opioid withdrawal symptoms, positive mood, and negative mood. Withdrawal symptoms were assessed with the Subjective Opiate Withdrawal Scale (SOWS; [31]). The scale includes withdrawal symptoms such as tremors, nausea, and perspiration. In our sample, Cronbach's α = 0.87. Scores of positive and negative mood were obtained using the Positive and Negative Affect Schedule (PANAS); a widely used scale with strong psychometric properties [32], [33]. This twenty-item scale yields separate, largely uncorrelated scores for positive (e.g., “excited,” “strong,” “interested”) and negative (e.g., “nervous,” “irritable,” “upset”) affect.

### Materials and Equipment

The study drug was naltrexone, approved by the U.S. Food and Drug Administration for treating narcotic dependency [34]. Naltrexone is an opioid-receptor antagonist with particular affinity for *mu*-opioid receptors [35]. It is orally absorbed, reaches peak plasma levels in approximately one hour, and has a half-life of about 4 hours [36]. The drug is a competitive antagonist, preventing active opioid molecules from docking at receptor sites. The major biologically active metabolite is 6-β-naltrexol, which has a half-life of from 12 to 18 hours [37]. In healthy volunteers, naltrexone is not associated with any significant adverse events [38], [39]. Naltrexone has been employed successfully in pain studies as an alternative to intravenously-administered naloxone [40].

Naltrexone and placebo capsules were compounded by the Campus Health Service Pharmacy at Arizona State University. Opaque gelatin capsules were filled with 50mg of naltrexone+190mg corn starch filler, or 240mg corn starch filler for placebo. Weights were determined on an Acculab VIR Electronic Balance with readability to 1mg and precision rated at 2mg. Capsules were placed in prescription vials, labeled “A” and “B,” with the identity of each vial's contents known only to the pharmacy manager. All study personnel were blinded to the contents of capsules. The identities of the capsules were revealed by the pharmacy manager at the conclusion of the study.

Hot and cold stimuli were presented with a Medoc TSA-II (Medoc Advanced Medical Systems; Durham, NC) and Peltier-type, 30×30mm thermode. The TSA-II allows for rapid heating and cooling with a continuous contact surface. A two-button peripheral device allowed participants to indicate when threshold or tolerance levels had been reached.

### Procedures

**Figure 1:**
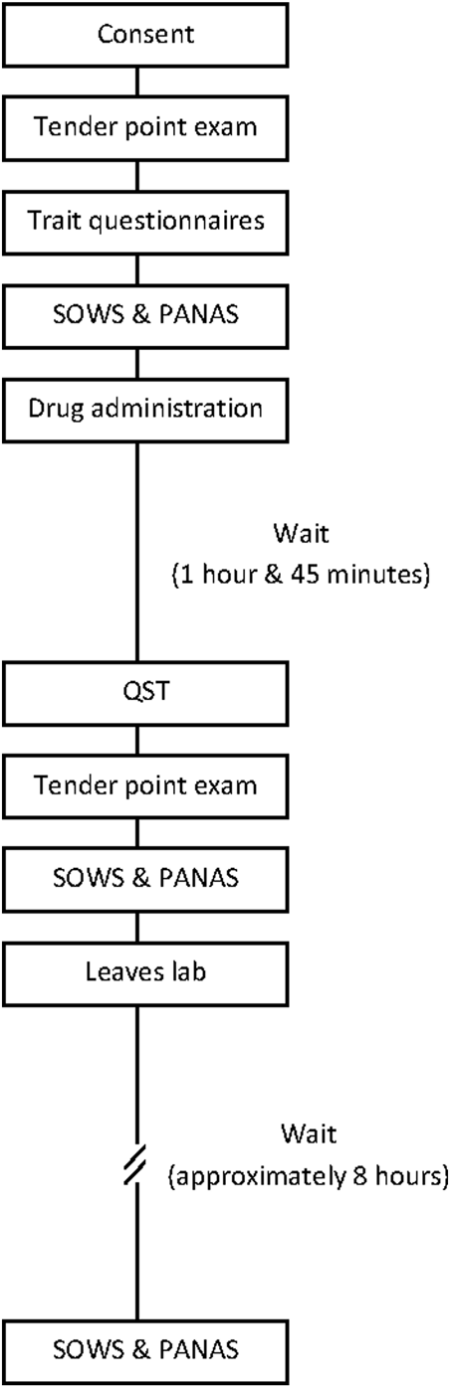
Flow diagram of study procedures. Study procedures conducted on each participant, once with naltrexone, and once with placebo. SOWS = Subjective Opioid Withdrawal Scale; PANAS = Positive and Negative Affect Schedule; QST = Quantitative Sensory Testing.

All procedures were carried out at the Community Health Services Clinic at ASU by research personnel blinded to the gelcaps' contents. Figure 1 shows the order of study procedures. Upon arrival and consenting, participants completed the baseline descriptive scales (visit #1 only), the mechanical pain test, and the SOWS and PANAS. Naltrexone or placebo was then orally administered by a research assistant, who then started a timer to run for one hour and 45 minutes. Drug randomization was performed using a computerized, random assignment generator. The delay before primary assessments allowed sufficient absorption of the drug. During the interim period, participants were trained on the thermal stimulator, and they underwent practice versions of all the tests. For the remainder of the time, participants were kept on the premises and allowed to read.

After the waiting period, participants underwent the three quantitative sensory tests for pain: heat pain threshold and tolerance, cold pain threshold and tolerance, and a second mechanical threshold. Each participant then completed the self-reported PANAS and SOWS, received $50.00, and was sent home with the instructions to complete an additional PANAS and SOWS, 7–8 hours after leaving the lab.

### Statistical analysis

We used SPSS version 17 to conduct all hypotheses tests. To compare fibromyalgia and control groups on the baseline measures, we used simple between-group (independent) *t*-tests. For all other tests (heat pain, cold pain, mechanical pain, withdrawal symptoms, and mood), we used linear mixed models. Subject ID was included as a random effect, and an unstructured covariance type was used for all analyses. Group (fibromyalgia versus control) and drug (naltrexone versus placebo) were included as fixed effects. For measures taken at multiple times throughout the day, time was included as an additional fixed effect.

## Results

Two individuals with a physician diagnosis of fibromyalgia did not meet the American College of Rheumatology [28] criteria for mechanical sensitivity, and were excluded from all analyses. The remaining 8 fibromyalgia participants all had an average daily pain level of at least 5 (on a 10-point scale). All fibromyalgia participants met widespread pain conditions (pain above and below waist, on both sides of the body, and in the axial skeletal region). No individuals in the control group met criteria for fibromyalgia. Independent sample *t*-tests revealed that the fibromyalgia group had higher FIQ scores than healthy controls (47.3 versus 10.5; *t*(16) = 7.6, *p* <.001), as well as more positive tender points (14.9 versus 1.1; *t*(16) = 11.1, *p*<.001), more general somatic complaints (1.5 versus 0.7, *t*(16) = 3.4, *p* = .004) and greater sensory sensitivity (3.3 versus 2.4; *t*(16) = 4.2, *p* = .001).

The effects of drug (naltrexone versus placebo) and group (fibromyalgia versus control) were tested on the three quantitative sensory tasks (see Table 1 for all means and standard deviations). Fibromyalgia patients had lower heat pain thresholds than controls (*F*(1,16) = 7.31, *p* = 0.016), marginally lower heat pain tolerance (*F*(1,16) = 4.2, *p* = 0.057), more sensitivity to cold pain onset (*F*(1,16) = 9.2, *p* = 0.008), and less tolerance for cold pain (*F*(1,16) = 8.5, *p* = 0.010). There were no main effects for drug, nor any drug×group interactions for heat or cold pain tests. We then tested the effects of naltrexone on mechanical pain sensitivity. Fibromyalgia patients had significantly lower mechanical pain thresholds than controls (*F*(1,16) = 187.04, *p*<.0005). A significant effect for drug emerged, with both groups showing increased thresholds (i.e., decreased pain sensitivity) during the naltrexone trial (*F*(1,16) = 5.48, *p* = .032). However, there were no significant interactions with group; fibromyalgia and control individuals reacted similarly to the drug administration.

**Table 1:** Drug and placebo means (SD) for all repeated-measures tests in Fibromyalgia and healthy controls.

	Fibromyalgia		Healthy Control	
	Drug	Placebo	Drug	Placebo
Heat threshold(°C)	41.4 (2.5)	41.6 (4.2)	44.2 (1.8)	44.3 (1.7)
Heat tolerance(°C)	44.8 (2.1)	45.2 (2.3)	46.5 (1.7)	46.7 (1.2)
Cold threshold (°C)	17.1 (5.7)	16.2 (7.3)	8.8 (6.6)	8.6 (4.6)
Cold tolerance(°C)	11.0 (5.4)	10.3 (6.8)	4.1 (4.2)	3.7 (4.3)
Pressure threshold (kg/cm2)				
Pre drug	5.6 (0.9)	6.4 (1.1)	9.7 (0.3)	9.9 (0.2)
Post drug	5.7 (1.0)	6.1 (1.0)	9.7 (0.3)	9.8 (0.3)
Opioid withdrawal				
Pre drug	19.5 (2.7)	20.1 (4.0)	15.7 (1.1)	16.0 (1.1)
Post drug	20.0 (3.7)	19.5 (3.8)	15.5 (1.1)	15.7 (0.8)
Home	23.1 (5.1)	20.1 (3.6)	17.4 (2.9)	15.9 (1.2)
Positive mood				
Pre drug	2.4 (0.9)	2.8 (0.9)	2.6 (0.7)	2.5 (0.7)
Post drug	2.2 (1.0)	2.3 (1.1)	2.2 (0.7)	2.4 (0.7)
Home	2.2 (1.0)	1.8 (0.8)	1.9 (0.6)	1.8 (0.6)
Negative mood				
Pre drug	1.3 (0.5)	1.2 (0.6)	1.0 (0.1)	1.0 (0.1)
Post drug	1.2 (0.2)	1.3 (0.6)	1.0 (0.0)	1.0 (0.2)
Home	1.5 (0.7)	1.3 (0.8)	1.3 (0.9)	1.0 (0.1)

**Figure 2:**
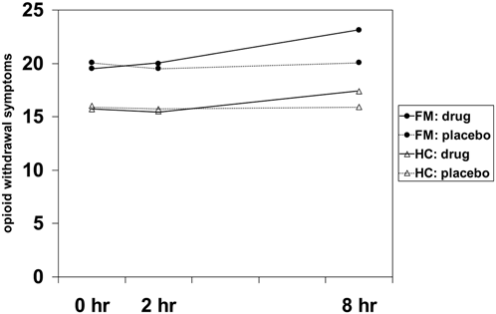
Effects of naltrexone on self-reported opioid withdrawal symptoms. Self-reported opioid withdrawal symptoms at 0 hours, 2 hours, and 8 hours (home measurement) after drug and placebo administration in Fibromyalgia patients (FM) and healthy controls (HC).

The effects of drug, group, and time (before capsule, 2 hours after capsule, and 8 hours after capsule) were tested on the following variables: opioid withdrawal symptoms, positive mood, and negative mood. For *opioid withdrawal symptoms* (Figure 2), a significant group effect was revealed (*F*(1,16) = 24.4, *p*<0.0005), with fibromyalgia patients reporting a significantly greater number of withdrawal-type symptoms. There was also a significant effect for time (*F*(1,16) = 6.7, *p* = 0.008), with symptoms increasing at the final measurement period (approximately 8 hours after drug administration). However, the drug×time interaction failed to reach significance (*F*(1,16) = 2.5, *p* = 0.116). Furthermore, there was no interaction between group and drug; patients and controls reacted similarly to the naltrexone administration. For *positive mood*, there was a significant drop over time (*F*(1,16) = 17.58, *p*<.0005). No other significant effects emerged. For *negative mood*, there were no significant effects for group, drug, time, nor any interactions.

## Discussion

In this study, we investigated the role of endogenous opioid peptides in fibromyalgia pain sensitivity. Fibromyalgia participants and age- and gender-matched controls showed similar responses to naltrexone, a potent opioid antagonist. We found no evidence of opioid dysregulation in fibromyalgia.

### Baseline comparison between fibromyalgia and healthy control groups

Many studies have identified elevated sensory, somatic and psychological symptoms in those with fibromyalgia; greater sensitivity to heat, cold, and mechanical pain [10], greater windup pain [21], greater incidence of affective disorders [41], general somatic complaints [42], cognitive impairment [43] and reproductive and sleep issues [44]. Fibromyalgia often overlaps with irritable bowel, chronic fatigue [45], and multiple chemical sensitivity syndromes [46]. Consistent with several previously reported group differences, we identified several baseline group differences. In addition to a self-reported higher sensitivity to a range of stimuli, and a greater number of somatic complaints than controls, fibromyalgia patients also had higher sensitivity to heat, cold, and mechanical pain. The wide variety of stimuli to which fibromyalgia individuals are sensitive suggests that a central nervous system amplification of signals may underlie fibromyalgia symptomotology [47], [48].

### Effects of naltrexone

Neither experimental hypothesis was supported by the results, as both the fibromyalgia and control groups responded similarly to naltrexone administration. Pain threshold and tolerance were not affected by opioid antagonism in either group. Overall, our findings agree with those from similar past experiments–fibromyalgia pain does not appear to be related to beta-endorphin dysregulation [19]–[21]. Interestingly, both groups experienced significantly *lower* mechanical pain sensitivity on the naltrexone-administration visit, contrasted with the placebo visit. Again, there was no divergence in response between fibromyalgia and control groups, further suggesting that the *mu*-opioid system is not a site of dysregulation.

Both groups exhibited increased opioid withdrawal symptoms over time. While Figure 2 suggests that this effect was most pronounced after naltrexone administration, the effect of drug on withdrawal symptoms was not statistically significant. This marginal effect may have been suppressed by a small sample size. Even so, the two groups showed no differential response to naltrexone. It is interesting that the effects of naltrexone may be observed so long after administration. The delayed effect of naltrexone has been previously reported [49], and may be due to the extended half-life of the 6-β-naltrexol metabolite [50], [51]. It is unknown if naltrexone would have produced changes in pain processing if measured at a later time-point.

Four methodological issues limit our interpretation of our results. First, the small sample size may not have provided adequate power for all tests. Observed effect sizes (mean standard differences) for within-group naltrexone effects, however, were very small, ranging from 0.05 to 0.19. Post-hoc power analyses revealed that, with alpha of 0.05 and beta of 0.80, a minimum of 220 participants per group would be needed to detect a significant effect. Therefore, for most tests, the nonsignificant findings were likely not due to inadequate sample size. Second, we did not collect information on menstrual phase, even though sensitivity to experimental pain is known to vary with fluctuating sex hormones [52]. The effect of menstrual phase was likely minimized as sessions were conducted no more than 72 hours apart. Third, participants' medications may have affected results from some tests. Our use of within-person statistics, and exclusion of those taking opioid medications, mitigates the impact of medication use. Fourth, only one dose of naltrexone was tested, and a larger dose may have produced larger changes in the dependent variables. We note, however, that 50mg is a typical dose for strongly blocking *mu*-opioid receptors [39], [53], [54], and larger doses may do more to extend the duration of action rather than increasing the completeness of the blockade [55].

Several opportunities for future studies exist. Beta-endorphins represent just one of a number of opioids, themselves a small part of all pain-related peptides. Other components of the peptide system of pain should be systematically investigated, including other opioid-peptides such as met-enkephalin [56], algesic opioid peptides such as nociceptin/orphanin FQ [57], nerve growth factor [58], calcitonin gene related peptide, substance P [59], and dynorphin A [60]. Further research may also utilize more sensitive measurement techniques, such as temporal summation produced by rapid heat taps.
